# Interleukin-6 Inhibitor-Induced Leukocytoclastic Vasculitis: A Case Report With a Literature Review

**DOI:** 10.1155/crii/8863357

**Published:** 2025-11-23

**Authors:** Tatevik Aloyan, Dinara Salimova, Ebrahim Mohamed, Hina Wazir

**Affiliations:** Department of Internal Medicine, Ascension Saint Joseph Hospital, 2900 N Lake Shore Dr, Chicago 60657, Illinois, USA

**Keywords:** case report, leukocytoclastic vasculitis, rheumatoid arthritis, tocilizumab

## Abstract

**Background:**

Leukocytoclastic vasculitis (LCV) is a known hypersensitivity reaction to biologic agents, often linked to tumor necrosis factor-α (TNF-α)inhibitors. We present a rare case of LCV in a patient receiving tocilizumab, a monoclonal antibody directed against interleukin-6 (IL-6)receptor.

**Case presentation:**

A 33-year-old Asian female with seropositive rheumatoid arthritis presented to the rheumatology clinic complaining of a new rash that started a few days after an infusion of tocilizumab. Her rheumatoid arthritis had been managed with upadacitinib for several years, which was discontinued due to persistent transaminitis. She was started on tocilizumab after a 4-month break from biologics. Following the first tocilizumab infusion, she recalled having transient fatigue and several red dots on her forearms and feet. A few days after the second infusion, she developed a purpuric rash on her lower extremities and forearms. Skin biopsy confirmed LCV. The rash resolved slowly in a month after discontinuation of tocilizumab and prescription of prednisone 20 mg daily. At her 3-month follow-up, the patient remained in remission, and her rheumatoid arthritis was uneventfully managed with abatacept.

**Conclusions:**

While most cases of biologic-associated LCV are induced by TNF-α inhibitors, only two known cases of tocilizumab-induced hypersensitivity vasculitis have been published in the literature. Our case represents only the third reported instance in the literature, highlighting the need to raise awareness of tocilizumab as a potential cause of leukocytoclastic vasculitis and the importance of prompt recognition and management.

## 1. Introduction

Leukocytoclastic vasculitis (LCV) is a type of small vessel vasculitis caused by immune complex deposition and neutrophilic infiltration of the vessel walls, leading to inflammation and tissue damage. It often occurs as a hypersensitivity reaction to various medications, infections, or systemic diseases. Commonly implicated drugs include antibiotics, nonsteroidal anti-inflammatory drugs (NSAIDs), biologic agents, selective serotonin reuptake inhibitors (SSRIs), diuretics such as furosemide, allopurinol, amiodarone, and metformin [1–3]. With the increasing use of targeted biologics in rheumatology, cases of drug-induced LCV have become more common, particularly with tumor necrosis factor-α (TNF-α) inhibitors [4]. Tocilizumab, a humanized monoclonal antibody targeting interleukin-6 (IL-6) signaling, is widely used to treat rheumatoid arthritis and other autoimmune conditions. Known hypersensitivity reactions include anaphylaxis and various skin reactions [5]. The American Academy of Allergy, Asthma, and Immunology (AAAAI) provides recommendations for evaluating and managing such reactions, including skin testing and desensitization protocols for tocilizumab [5]. Although tocilizumab is increasingly used for autoimmune diseases, it is rarely associated with LCV. A systematic review identified only one reported case of tocilizumab-induced cutaneous vasculitis, underscoring how uncommon this adverse effect is [4]. This case report aims to add to the limited evidence by describing a patient with seropositive rheumatoid arthritis who developed LCV following the initiation of tocilizumab therapy.

## 2. Case Presentation

**Figure 1:**
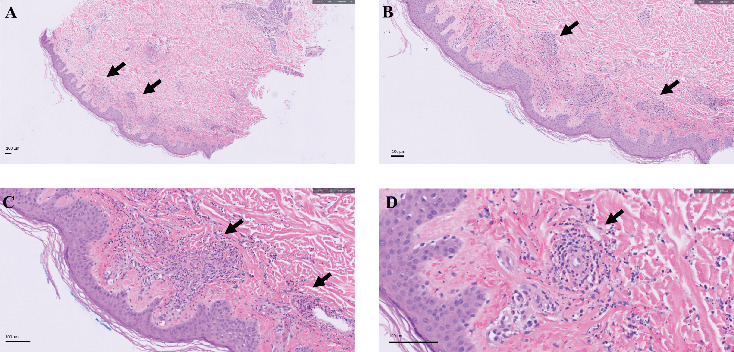
Histopathological examination with hematoxylin and eosin (H&E) staining reveals a perivascular neutrophilic infiltrate, accompanied by fibrinoid necrosis. These findings are demonstrated at ×5 (A), ×10 (B), ×20 (C), and ×40 (D) magnifications. Arrows highlight the areas of infiltrates.

A 33-year-old Asian female with seropositive rheumatoid arthritis and no concomitant medical history presented to the rheumatology clinic with a new rash that started 2 days after infusion of tocilizumab. She had been diagnosed with RA 7 years ago and was managed with upadacitinib, a Janus kinase inhibitor, for the past 2 years. This was stopped due to persistent mild transaminitis. After discontinuing the upadacitinib, she had a 4-month break from biologics with a resolution of her transaminitis. She was managed with prednisone 10 mg daily during the window period and subsequently was started on tocilizumab. Following the first tocilizumab infusion, she recalled having transient fatigue and several red dots on her forearms and legs. Within 2 days of the second infusion, she developed a rash on the same sites. She denied fever/chills, chest pain, shortness of breath, abdominal pain, or urinary changes. The physical examination revealed palpable, petechial, and purpuric lesions over her legs, forearms, and shoulders. Laboratory studies were significant with elevated erythrocyte sedimentation rate, C-reactive protein, positive perinuclear anti-neutrophil cytoplasmic antibodies (p-ANCA), and normal complement 3 and 4 levels [Table 1]. Her complete blood count, metabolic panel, and urinalysis were unremarkable. The skin biopsy showed perivascular neutrophilic infiltrates with leukocytoclasia and immune complex deposition in the vessel walls, confirming LCV [Figure 1]. The rash resolved slowly in a month following discontinuation of tocilizumab and initiation of prednisone 20 mg daily. At her 3-month follow-up, she remained with no recurrence of the rash. She underwent the Prism RA test, predicting an inadequate response to TNF-α inhibitors and was uneventfully managed with abatacept, a CD80/86 inhibitor, and a slow taper of prednisone.

## 3. Discussion

LCV can occur with a wide variety of medications, including antibiotics, NSAIDs, biologics, SSRIs, furosemide, allopurinol, amiodarone, and metformin [1–3]. A literature review was conducted using PubMed and Google Scholar databases. It revealed only two case reports of tocilizumab-induced hypersensitivity vasculitis, while most cases were linked to TNF-α inhibitors [5–7]. In a systematic review by Ak et al. [4], 123 cases with a biologic agent-associated cutaneous vasculitis were reviewed. The histopathology revealed LCV in 73% of cases (*n* = 90). TNF-α inhibitor-related cutaneous vasculitis was reported in 59.5% of cases (*n* = 73), while tocilizumab was responsible for only one case (0.8%) [4]. In the systematic review by da Silva et al. [8], 89 cases of biologic agent-induced cutaneous vasculitis were reviewed. The most common type of vasculitis was nonspecific LCV (42%, *n* = 37), followed by ANCA-associated vasculitis (18%, *n* = 16). The most common involved agents were TNF-α inhibitors in 81% of cases (*n* = 72), including infliximab in 30%, adalimumab in 22.5%, etanercept in 20%, certolizumab, and golimumab in 4.5% and 3.4%, respectively. Tocilizumab was reported in only one case (1.1%) [8].

Interestingly, the single case of tocilizumab causality in these two systematic reviews was the same case reported by Sakaue et al. [7], which was one of the two case reports we found from the PubMed search initially, making our article the third case described of this kind.

In our case, the extensive evaluation mentioned above did not reveal evidence of infection, autoimmune flare, or other common triggers of LCV, pointing toward a drug-induced etiology.

Additionally, the Naranjo Adverse Drug Reaction Probability Scale was used to assess the likelihood of tocilizumab causality, yielding a score of five, indicating a probable adverse drug reaction based on the temporal sequence, clinical improvement upon withdrawal, and a low likelihood of an alternative clinical explanation [9, 10].

While hypersensitivity reactions to tocilizumab are rare, the AAAAI suggests that the inhibition of the IL-6 pathway may lead to immune dysregulation in susceptible individuals [5]. This possibility is supported by the observation that tocilizumab treatment increases serum levels of IL-6 and soluble IL-6 receptor (sIL-6R) [11]. Also, tocilizumab binds to both sIL-6R and membrane-bound IL-6 receptor (mIL-6R), effectively blocking IL-6 from activating its receptors. However, it does not interfere with the alternative receptors that IL-6 could bind under certain conditions [12, 13]. This interplay might contribute to the development of hypersensitivity reactions, including LCV. For example, abnormally elevated concentrations of IL-6 could possibly saturate the IL-11 receptors, leading to dysregulated STAT3 activation, promoting chronic inflammation and autoimmunity [13, 14]. However, this remains a hypothetical explanation based on limited mechanistic evidence and has not been directly confirmed in clinical settings.

Prompt recognition and discontinuation of the offending drug are crucial for resolving LCV. In this case, the patient's rash improved after tocilizumab was discontinued and corticosteroid therapy was initiated. This approach aligns with current recommendations for managing drug-induced hypersensitivity vasculitis [5]. Transitioning the patient to abatacept allowed successful management of her rheumatoid arthritis without recurrence of vasculitis.

This case illustrates the potential of tocilizumab to induce LV and highlights the need for clinical vigilance and timely intervention, particularly as the therapeutic use of tocilizumab expands. Further research is necessary to understand the mechanisms behind drug-induced LCV and to identify risk factors that may predispose patients to this complication.

## Figures and Tables

**Table 1:** Relevant laboratory results.

Test	Result	Reference range
White blood cells (WBCs)	7300 cells/μL	4000–11,000 cells/μL
Platelets	160,000 cells/μL	150,000–450,000 cells/μL
Erythrocyte sedimentation rate (Westergren)	**56 mm/h (H)**	0–32 mm/h
C-reactive protein, quant	**13 mg/L (H)**	0–10 mg/L
Blood urea nitrogen (BUN)	12 mg/dL	6–20 mg/dL
Creatinine, serum	0.85 mg/dL	0.57–1.00 mg/dL
Complement C3	102 mg/dL	90–180 mg/dL
Complement C4	16 mg/dL	10–40 mg/dL
Anti-MPO antibodies	0.9 units	0.0–0.9 units
Anti-PR3 antibodies	<0.2 units	0.0–0.9 units
Cytoplasmic anti-neutrophil cytoplasmic antibodies (C-ANCA)	<1:20	Neg: <1:20 titer
Perinuclear anti-neutrophil cytoplasmic antibodies (P-ANCA)	**1:160 (H)**	Neg: <1:20 titer
Atypical pANCA	<1:20	Neg: <1:20 titer

*Note:* The abnormal values are shown in bold for easier visualization.

## Data Availability

The data that support the findings of this study are available from the corresponding author upon reasonable request.

## Nomenclature

AAAAI: American Academy of Allergy, Asthma and Immunology
CD: Cluster of differentiation
CRP: C-reactive protein
ESR: Erythrocyte sedimentation rate
H&E: Hematoxylin and eosin
IL-6: Interleukin-6
LCV: Leukocytoclastic vasculitis
mIL-6R: Membrane-bound IL-6 receptor
p-ANCA: Perinuclear antineutrophil cytoplasmic antibodies
RA: Rheumatoid arthritis
sIL-6R: Soluble IL-6 receptor
TNF-α: Tumor necrosis factor-α.

## References

[1] Baigrie D., Crane J. S. (2025). Leukocytoclastic Vasculitis. 2023 Aug 8. *StatPearls [Internet]*.

[2] Betsikos A., Mousia M., Simopoulou E. (2024). Cutaneous Small-Vessel Vasculitis Induced by Escitalopram: A Case-Based Brief Review of the Literature. *Cureus*.

[3] Nishioka H., Fujita S., Hara S. (2024). Cefazolin-Induced Leukocytoclastic Vasculitis. *Postgraduate Medical Journal*.

[4] Ak T., Durmus R. B., Onel M. (2023). Cutaneous Vasculitis Associated With Molecular Targeted Therapies: Systematic Review of the Literature. *Clinical Rheumatology*.

[5] Broyles A. D., Banerji A., Barmettler S. (2020). Practical Guidance for the Evaluation and Management of Drug Hypersensitivity: Specific Drugs. *The Journal of Allergy and Clinical Immunology: In Practice*.

[6] Hu Y.-Q., Chen X., Zhang J.-Z. (2022). Leucocytoclastic Vasculitis-Like Reaction Induced by Tocilizumab in a Patient With Rheumatoid Arthritis. *Dermatologic Therapy*.

[7] Sakaue S., Sumitomo S., Kubo K., Fujio K., Yamamoto K. (2014). Tocilizumab-Induced Leucocytoclastic Vasculitis in a Patient With Rheumatoid Arthritis. *Rheumatology*.

[8] da Silva Cendon Duran C., da Paz A. S., Barreto Santiago M. (2022). Vasculitis Induced by Biological Agents Used in Rheumatology Practice: A Systematic Review. *Archives of Rheumatology*.

[9] Pradhan P., Corbin S., Todkar S. (2025). Serious Adverse Events: A Replicability and Validation Study of Naranjo Causality Assessment Tool in a Canadian Clinical Setting. *Journal of Evaluation in Clinical Practice*.

[10] Deutscher B., De Guzman K., La Caze A., Falconer N. (2024). A Scoping Review of the Clinical Utility of Adverse Drug Reaction Causality Analysis Tools for use in the Hospital Setting. *Expert Review of Clinical Pharmacology*.

[11] Nishimoto N., Terao K., Mima T., Nakahara H., Takagi N., Kakehi T. (2008). Mechanisms and Pathologic Significances in Increase in Serum Interleukin-6 (IL-6) and Soluble IL-6 Receptor After Administration of an Anti-IL-6 Receptor Antibody, Tocilizumab, in Patients With Rheumatoid Arthritis and Castleman Disease. *Blood*.

[12] Mihara M., Kasutani K., Okazaki M. (2005). Tocilizumab Inhibits Signal Transduction Mediated by Both mIL-6R and sIL-6R, but not by the Receptors of Other Members of IL-6 Cytokine Family. *International Immunopharmacology*.

[13] Weitz H. T., Ettich J., Rafii P. (2025). Interleukin-11 Receptor Is an Alternative α-Receptor for Interleukin-6 and the Chimeric Cytokine IC7. *The FEBS Journal*.

[14] Rose-John S., Jones S. A. (2025). More and More Pleiotropy Within the IL-6 Family of Cytokines. *The FEBS Journal*.

